# A dataset from a population-wide-scale survey of academics in Iceland on barriers to collaboration with industry and community

**DOI:** 10.1016/j.dib.2023.109538

**Published:** 2023-09-07

**Authors:** Verena Karlsdottir, Magnus Thor Torfason

**Affiliations:** a University of Akureyri, Norðurslóð 2, Akureyri 600, Iceland; b University of Iceland, Sæmundargata 2, Reykjavik 102, Iceland

**Keywords:** University-industry collaboration, Third mission, Entrepreneurial university, Academic engagement, Academics, Higher education

## Abstract

In early 2021, a quantitative survey was conducted among academics in Icelandic universities to gather information about their involvement in Third Mission (TM) activities and barriers to collaboration [1]. The target group consisted of all academics working at any of the seven Icelandic universities. The majority of participants (*n* = 674) were affiliated with the University of Iceland, while the remaining (*n* = 360) were associated with other universities. The survey was designed based on previous studies on barriers to university-industry collaboration [2]. Before it was administered, it underwent a pre-test phase involving various academics and university staff members. Email addresses of potential participants were obtained from the universities’ public websites. The survey was conducted using QuestionPro over a period of 21 days, with two reminders being sent following the initial invitation to participate. A total of 183 responses were collected, although not all participants completed the entire questionnaire. Consequently, the response rate amounted to 17.7%. It is worth noting that the study is a census, i.e., it targeted all academics in Iceland that satisfied the participation criteria rather than targeting only a sample of the population. Furthermore, the data extends its focus to academic disciplines that have previously received limited attention in third mission research.

Specifications Table

**Table:** 

Subject	Social Sciences – Education
Specific subject area	Collaboration activities of academics; third mission activities in educational institutions; entrepreneurial universities
Data format	Raw
Type of data	Table (Excel) Survey responses, arranged in a rectangular data set of variables (questions) by observations (participants).
Data collection	Data were collected with an online survey in March 2021 among all academics in Icelandic universities. Academics at all seven Icelandic universities received a link via email, inviting them to participate, followed by two reminders. Question items on barriers to collaboration were derived from Goduscheit and Knudsen (2015).
Data source location	· Institution: University of Iceland · City/Town/Region: Reykjavik · Country: Iceland · Latitude and longitude (and GPS coordinates, if possible) for collected samples/data: 64.123753, -21.926866
Data accessibility	Repository name: Gagnís (DATICE) Data identification number: 10.34881/KYAP6J Direct URL to data: https://dataverse.rhi.hi.is/dataset.xhtml?persistentId=doi:10.34881/KYAP6J
Related research article	Karlsdottir, V., Torfason, M. T., Edvardsson, I. R., & Heijstra, T. M. (2023). Barriers to academic collaboration with industry and community: Individual and organisational factors. Industry and Higher Education (Advanced online publication). https://doi.org/10.1177/09504222231173953

## 1 Value of the Data

• The data help to identify barriers to academic collaboration, informing development of strategies and policies to improve interaction within institutions and increasing research productivity.
• The data can benefit multiple stakeholders: academic institutions can create effective interventions and support mechanisms; policymakers can use insights to shape education and research policies; academic professionals gain increased awareness of collaboration challenges.
• Further statistical analyses of this data, including through pooling with other comparable datasets, can reveal insights into academic collaboration dynamics, including identifying key influencing variables and understanding their relationships.
• Longitudinal analysis of the data allows monitoring of collaboration barriers over time, helping to evaluate the effectiveness of interventions and changes in institutional policies.
• The data, when compared with international datasets, can shed light on universal or region-specific barriers to collaboration, enriching understanding of contextual factors affecting academic practices.

## 2 Data Description

The data and accompanying metadata are available through the Gagnís Dataverse of the School of Social Science/Social Science Research Institute (University of Iceland) [3]. The survey data itself is available in *barriers_data.tab* which can be downloaded in multiple formats, including for Stata, R, and as a tab-delimited text file.

Metadata is provided in several files. In *barriers_metadata_01_measurements.pdf*, we present detailed information about description of measurements, and how answer options were coded in the dataset. In *barriers_metadata_02_codebook.pdf,* we report variable names, a brief variable description, and response options including variable frequencies. The file *barriers_metadata_03_adjustements.pdf* provides information about adjustments to survey items that were adapted from Goduscheit and Knudsen [2]. The above metadata files, 01-03, are also available in text format in the *barriers_metadata.tab* file, which can be downloaded in the original Excel format (three sheets), and in tab-delimited or R format. The file *barriers_metadata_04_methodology.pdf*, provides a description of the study implementation, and finally, the full survey text is available in *barriers_metadata_05_survey.pdf* both in English and Icelandic.

Table 1 provides the characteristics of the dataset's sample.

**Table 1:** Sample characteristics.

	N	%
**Gender**		
Male	66	44
Female	84	56
**Age**		
≤ 49	56	36.6
50–59	49	32
≥ 60	48	31.4
**Position**		
Non-professor (Junior Faculty)	80	53.7
Professor (Senior Faculty)	70	46.7
**Discipline**		
STEM and Health	59	39.1
Other disciplines	92	60.9
**Size of university**		
Small	65	35.5
Large	118	64.5
**Experience outside academia**		
No	108	69.7
Yes	47	30.3
**Number of articles co-authored with non-academics**		
None	76	49
One article	36	23.3
Two or more	43	27.7
**Application for funding together with industry/public organisation**		
Never	74	41.1
Rarely	35	19.4
Occasionally	33	18.3
Often	20	11.1
Very often	18	10
**Formal R&D collaboration such as joint or contract research**		
Never	39	21.8
Rarely	33	18.4
Occasionally	40	22.3
Often	36	20.1
Very often	31	17.3

The study included the total population of academics from all seven universities in Iceland, totaling 1,034 individuals holding positions as adjunct lecturers, assistant professors, associate professors, or professors. Out of this population, 183 responses were collected, resulting in a response rate of 17.7%. The gender distribution among participants showed that approximately 56% were women, while 36.6% were 49 years old or younger, and 31.4% were 60 years or older. The data have been validated through factor analyses and reliability measures; further information on this procedure can be found in the related research article of Karlsdottir et al. [1].

Regarding the distribution across academic disciplines, the majority of respondents were affiliated with the School of Social Sciences (26.5%) and the School of Natural Sciences (25.8%). The Agricultural University of Iceland had the fewest responses (2.6%), and there were no responses from the University of Arts. In order to maintain better anonymity among participants, two categories were created: science, technology, engineering, mathematics (STEM) and health sciences (39.1%), and all other sciences (60.9%).

In terms of academic positions, the highest number of responses came from full professors (46.5%), followed by associate professors (22.5%) and assistant professors (24%). The lowest number of responses came from adjunct lecturers (6.5%). Also here, to anonymize the data, we created a dummy variable of two categories: professor (47.7%) and no-professor (53.7%).

Descriptive statistics for barriers to collaboration can be seen in Table 2, including, number of observations (n), minimum and maximum values, means, and standard deviations.

**Table 2:** Descriptive statistics for barriers to collaboration.

Variable	N	Min.	Max.	Mean	St.dev.
Teaching requires too much time	174	1.00	5.00	3.37	1.20
My research focus is not interesting enough for companies/organisations	166	1.00	5.00	2.45	1.22
Difficult to get informed about research activities of companies and other organisations (confidentiality)	129	1.00	5.00	2.29	1.04
Difficult to find an appropriate and relevant partner among companies/organisations	146	1.00	5.00	2.49	1.13
Scientific independence impaired	140	1.00	5.00	2.04	1.21
Hindrance to academic publication activities	143	1.00	5.00	2.55	1.23
Negative effect on academic freedom	147	1.00	5.00	2.17	1.18
Pressure for short-term research from companies/organisations	127	1.00	5.00	2.17	1.24
Negative effect on long-term research	133	1.00	5.00	2.11	1.17
Lack of qualified staff on the part of companies/organisations	128	1.00	5.00	2.25	1.26
Lack of technical facilities on the part of companies/organisations	115	1.00	5.00	1.95	1.11
Lack of interest in scientific projects on the part of companies/organisations	137	1.00	5.00	2.82	1.26
Lack of knowledge among the companies about the potential in collaboration with the universities	138	1.00	5.00	2.81	1.25
Approach of my department's staff is not entrepreneurial enough	131	1.00	5.00	1.93	1.07
Lack of possibilities to commercialize my research findings (e.g. administrative support)	129	1.00	5.00	2.22	1.23
The companies/organisations have different ideas on costs, time and/or productivity	132	1.00	5.00	2.66	1.18
R&D budgets of potential business partners are too low	128	1.00	5.00	2.96	1.26
Resource-intensive administrative and approval procedures, legal restrictions within your organisation	128	1.00	5.00	2.26	1.17
Lack of project administration support on the part of the academic institution	128	1.00	5.00	2.42	1.23
Property right problems	108	1.00	5.00	1.93	1.13

## 3 Experimental Design, Materials and Methods

The third mission (TM) of universities, which involves knowledge exchange and collaboration with industry and society, plays a crucial role in advancing societal impact and fostering innovation [4]. However, several barriers can impede academics from actively participating in TM activities. This study sought to identify and understand these barriers among academics from all seven Icelandic universities. Thereby, a quantitative survey was conducted in early 2021 to explore barriers that academics face in engaging in TM activities across all seven Icelandic universities. The study targeted adjunct, assistant, associate, and full professors to gain insights into their engagement challenges.

In this study, the survey explored barriers associated with the collaboration activities of academics spanning a 3-year timeframe from 2018 to 2020. To assess these barriers comprehensively, the research questionnaire incorporated items adapted from a Nordic survey conducted by Goduscheit and Knudsen [2]. The original survey by Goduscheit and Knudsen [2] focused on barriers to collaboration between universities and small and medium-sized enterprises (SMEs). By adapting these items, the present study aimed to gain valuable insights into the hindrances that academics face in their collaborative endeavours during the specified period.

Survey questions further inquired about academic rank, discipline, experience outside academia, and publication and research activities. These questions were adapted from Bekkers and Freitas [5], Boardman and Ponomariov [6], Bourelos et al. [7], D'Este and Patel [8].

Unlike traditional sampling methods, this study targeted the entire population of academics from all seven Icelandic universities consisting of 1,034 academics. By doing so, it aimed to be inclusive of academic disciplines that might have been overlooked in previous TM studies, thereby enhancing the generalizability of its findings.

Before launching the main survey, a pre-test was conducted with academics and university staff to refine the research instrument. Email addresses were collected from the publicly available websites of the universities. Two identical surveys were distributed simultaneously: one to the University of Iceland and the other to the remaining six universities. Eventually, the responses from both surveys were combined into a single data file, enabling differentiation between a medium-sized university with over 10,000 students (University of Iceland) against smaller, more specialised universities with less than 10,000 students. The survey was administered in March 2021 using the online platform QuestionPro and remained open for 21 days. To improve response rates, two reminder emails were sent to the participants. Ultimately, 183 responses were collected, yielding a response rate of 17.7%. Although the response rate was not high, it was considered acceptable for further analysis.

Upon completion of the data collection phase, all information was downloaded and integrated into the R software for analysis. Data cleaning procedures were performed to remove irrelevant or potentially identifiable details, such as time stamps, IP addresses, and geolocation.

A non-response analysis was conducted to examine the disparities between early and late responses. The data demonstrated no statistically significant differences in demographic characteristics between these two response groups.

The 20 items on barriers to collaboration were adapted from Goduscheit and Knudsen (2015) and presented as declarative statements, prompting respondents to indicate their level of agreement on a 5-point Likert scale spanning from 'No importance' to 'High importance,' along with an option for 'Does not apply.'

The variables included measurements encompassing individual, inter-organisational, and intra-organisational domains. At the individual level, Gender was quantified as a binary variable (1 = male, 0 = female), while Age was categorized into three groups (49 years or younger; 50 – 59 years; 60 years or older).

The intra-organisational variables, centered within the academic institution, predominantly relate to academic position which is dichotomized as ‘Senior Faculty’ (i.e., full Professor rank, coded as 1) and ‘Junior Faculty’ (i.e. all non-professor academic positions, coded as 0). Academic discipline is encoded as a binary variable, distinguishing STEM and Health disciplines (1) from other fields (e.g., Social Sciences, Humanities, Education, Agriculture) (0), following the approach of Huyghe and Knockaert [9].

The ‘Experience outside academia’ variable denotes whether academics possessed prior experience beyond academic settings (‘Yes’, 1) or not (‘No’, 0), such as in companies or public organisations.

The ‘Number of articles co-authored with non-academics’ variable gauged whether academics had participated in article authorship or co-authorship with non-academic collaborators. Response options encompasses ‘None’ (0), ‘One’ (1), and ‘Two or more’ (1).

The prevalence of industry-focused research is captured through the frequency of endeavours such as ‘Application for funding together with industry/public organization’ and ‘Formal research and development (R&D) collaborations, such as contract research or joint research projects’. These were evaluated on a 5-point Likert scale spanning from ‘Never’ (1) to ‘Very often’ (5).

Lastly, the inter-organisational variable, ‘Size of university’, contrasts a medium-sized university with over 10,000 students (‘Large’, 1) against smaller institutions with less than 10,000 students (‘Small’, 0).

To unveil overarching constructs from reported collaboration barriers, a Principal Component Analysis (PCA) [10] was employed. Reliability of the scale was assessed through Cronbach's α. The PCA employed varimax rotation and Kaiser normalization. These tests demonstrated the variables to be generally reliable [1].

These data are suitable for further analysis, for example through pooling information with data collected in other past or future studies, and is aided by metadata that provides the exact wording of questions in this study in comparison with a prior study [2]. This includes combining the data with similar sets from other sources, which can uncover valuable insights concerning the dynamics of academic collaboration. Data pooling can thereby enhance sample size and statistical power. Globally, data aggregation supports cross-country models, fostering comprehension of key distinctions [11].

## 4 Limitations

It is important to acknowledge that the responses may not be entirely representative of the total population of academics in Iceland. In the overall population, approximately 57.5% of academic staff members are male. Similarly, the distribution of positions differs, with 41% being full professors, 19% associate professors, and 26% assistant professors.

Due to the low response rate, it was decided to create two groups regarding academic disciplines, one comprising STEM and health sciences and the other comprising all other disciplines. Further, academic position has been reduced to two groups (“professor” and “non-professor”).

## Ethics Statement

The authors declare that they comply with all the guidelines given by the Science Ethics Committee at the University of Iceland. Informed consent was obtained from all the participants, and all the data were anonymized.

## CRediT authorship contribution statement

**Verena Karlsdottir:** Conceptualization, Methodology, Data curation, Investigation, Visualization, Validation, Writing – original draft. **Magnus Thor Torfason:** Methodology, Data curation, Software, Validation, Writing – review & editing.

## Data Availability

barriers_data in brief for TM (Original data) (Dataverse).
